# The influence of high-speed rails on urban innovation and the underlying mechanism

**DOI:** 10.1371/journal.pone.0264779

**Published:** 2022-03-04

**Authors:** Yue Lu, Siying Yang, Jian Li

**Affiliations:** 1 School of Business, Jilin University, Changchun, China; 2 Centre for China Public Sector Economy Research and Economics School, Jilin University, Changchun, China; 3 Institute for Advanced Economic Research, Dongbei University of Finance and Economics, Dalian, China; Northeastern University (Shenyang China), CHINA

## Abstract

Innovation is intrinsically dependent on the construction of local infrastructure. Using panel data on 285 cities in China, we empirically examined the impact of high-speed rails on urban innovation and the mechanism underlying this effect. We found that high-speed rails significantly increase urban innovation. In our analysis, high-speed rails were found to increase the agglomeration of innovation factors, including population and investment, which in turn increase urban technological innovation. The agglomeration of investment factors brought about by high-speed rails is the main source of the improvement in urban innovation. Through the use of a spatial panel model, we found that high-speed rails promote knowledge dissemination and technology spillovers among the cities along high-speed railways, thus improving their innovation levels. However, the existing effects of high-speed rails on innovation exhibit spatial heterogeneity. We confirmed the effect of high-speed rails on innovation and explored the mechanism underlying this effect by considering the effects of factor agglomeration and knowledge spillovers. Our conclusions can be used as a resource by policymakers to stimulate knowledge and technology diffusion, which in turn cultivates and stimulates urban innovation.

## Introduction

Innovation activities within cities are intrinsically dependent on the local infrastructure [1], particularly on the transportation and communication infrastructure [2–4]. Although it is possible to conduct conventional innovation-related transactions, communicate and cooperate through the internet or over the phone, certain highly confidential innovative activities continue to rely on face-to-face communication among R&D workers for the dissemination and sharing of knowledge [5]. The flow of knowledge that occurs with modern information technology as the carrier cannot fully replace the flow of scientific researchers that occurs with the transportation infrastructure as the carrier. The compression of space and time induced by high-speed rails has made it more convenient for humans to travel in order to engage in innovative activities.

Since 1978, China’s railway mileage has nearly doubled. By 2016, the total mileage of China’s high-speed rail exceeded 22,000 kilometers, accounting for more than 60% of the total high-speed rail mileage in the world, and covered 177 prefecture-level cities [6]. During the same period, China embraced rapid increases in scientific and technological innovation. For example, the number of patent applications that the Chinese government has authorized has increased by more than 20% annually in recent years [4]. Since 2013, China has consistently ranked first in the world in terms of the number of patent applications for inventions. Is there an inherent relationship between the development of high-speed rails and urban innovation? The rapid development of China’s high-speed rail system and China’s great achievements in scientific and technological innovation provide a credible background for this study. Our research examines the influence of high-speed rails on urban innovation and the mechanism underlying this relation.

Previous research has identified the effect of transportation infrastructure on innovation. For example, Wang et al. (2018) found that highway density is significantly positively correlated with enterprise innovation, and highways can promote enterprise innovation by expanding enterprise market shares and accelerating knowledge spillovers [4]. Other studies focus on the effect of high-speed rails on innovation. For example, Zeng et al. (2021) examined the impact of high-speed rails on urban innovation activities and found that high-speed rails promote such activities through, for example, increasing the scale of innovation input and output [7]. Chen (2012) also confirmed the impact of high-speed rails on innovation by examining the impact of high-speed rails on energy consumption [8]. Lin (2017) found that high-speed rails promote industrial agglomeration, which in turn promotes urban innovation [9].

In summary, the existing literature has paid attention to the effect of high-speed rails on innovation. Our research differs from the existing studies in the following ways. First, instead of using the number of patent applications as an indicator of urban innovation levels, we use the urban innovation index. This index was calculated with the patent renewal model and can ease concerns regarding the heterogeneity in patent quality and value. Therefore, the urban innovation index is a more scientific indicator for measuring the level of urban innovation [10]. Second, unlike Zeng et al. (2021) and Lin (2017), who study the mechanisms of high-speed rails on urban innovation from the perspectives of the level of openness to trade, industrial agglomeration and synergy within cities [7, 9], we explore the mechanism that drives the impact of high-speed rails on urban innovation in terms of factor agglomeration, which enriches the research on the effect of high-speed rails on innovation. A spatial panel econometric model is used to analyze the differences in the innovation spillovers in cities with a high-speed rail before and after the opening of the high-speed rail to verify its role in the formation of innovation networks. Our research also helps to clarify why high-speed rails promote urban innovation; that is, we clarify the role played by knowledge spillovers between high-speed rail cities, which are not considered in previous empirical studies.

## Literature review and hypothesis

Existing research has focused primarily on the economic effects of the operation of high-speed rails. Ahlfeldt and Feddersen (2018), Wang and Ni (2016), Liu and Li (2017), and Yao et al. (2019) all found that high-speed rails significantly improved urban economic growth [11–14]. The underlying mechanisms include the following. 1) High-speed rail networks improve economic efficiency. For example, Shi et al. (2018) found that increasing railway speeds ensures market competition, improves the allocation of resources, and promotes the technological progress of enterprises located along railway lines [15]. 2) High-speed rails lead to more efficient factor reallocations. Du and Peng (2017) found that the opening of high-speed rails attracts more talent for regional development, which increases economic growth via the accumulation of human capital [16]. 3) High-speed rails increase the level of industrial agglomeration. For example, Li and Sun (2017) argued that high-speed rails promote regional manufacturing agglomeration [17]. Deng et al. (2017) found that high-speed rails also significantly promote the agglomeration of urban services [18]. 4) High-speed rails lead to structural adjustments as part of regional development. Wang and Ni (2016) found that the opening of a high-speed rail leads to increases in investment and that the variations in investment flows alter the regional industrial structure and affect growth [12]. Zhang (2017) found that high-speed rails improved the value added of the secondary industry in county-level cities and promoted urban economic growth [19].

In general, the construction of high-speed rails has profoundly affected local regional economic development. However, the construction of high-speed rails is not always a win–win situation [20, 21]. The impact of high-speed rails on the development of central and peripheral cities is unequal. For example, Qin (2017) found that the opening of a high-speed rail increases economic activity in central cities but also intensifies the siphoning of resources away from peripheral cities, which has a negative impact on the economic development of those cities [22]. Similarly, Coto-Millan et al. (2007) found that high-speed rails have promoted urban development in central Europe but have had a negative impact on the economic development of Spain, Portugal, and other peripheral areas [23]. Li and Xu (2018) also found that high-speed rails promote the development of the service industry in central areas (Tokyo Metropolitan Area) but lead to the shrinkage of the service industry in peripheral areas [24]. Using panel data on Chinese cities, Bian et al. (2018) found that the construction of high-speed rails is polarizing: the acceleration in factor flows has widened the development gap between central and peripheral cities [25]. The main reason why high-speed rails aggravate regional economic imbalances is that the opening of a high-speed rail facilitates the flow of economic factors toward central cities and the agglomeration of those factors within central cities, which increases the siphoning of resources by central cities away from peripheral cities [26].

Previous research has primarily analyzed the impact of high-speed rails on urban economic growth from the perspectives of efficiency enhancement, factor flows, industrial agglomeration, and structural adjustment effects, while less attention has been given to the impact of high-speed rails on urban innovation. Scientific innovation requires the construction of better urban infrastructure, and cities with a higher capacity for innovation are also the cities with more efficient transportation infrastructure.

As an important instrument for economic growth, the opening of a high-speed rail profoundly impacts urban innovation by strengthening intercity linkages through the construction of infrastructure. The development of a high-speed rail facilitates scientific innovation and accelerates the clustering of innovation resources, which can effectively improve the efficiency and level of innovation within a city.

First, regarding efficiency enhancements, high-speed rails can meet the increasing demand for (faster and better) travel within an urban population, which accelerates the flow of innovative elements, optimizes resource allocation, reduces the frictions in disseminating knowledge and technology over space and time, and improves the efficiency of the allocation of innovative resources.

Second, in terms of the agglomeration of human capital, suitable infrastructure layouts are instrumental in attracting talent and generating high-quality growth [27]. Debrezion et al. (2011) found that the opening of a high-speed rail led to the agglomeration of the population within cities [28]. Du and Peng (2017) also found that the opening of a high-speed rail attracted more senior-level talent in the development of the city [16]. High-speed rails create convenient intercity linkages that lure high-quality workers. Population agglomeration driven by the opening of a high-speed rail has three effects on urban innovation. 1) The concentration of high-quality talent leads to improvements in innovative efficiency due to the scale effect. 2) High-speed railways increase urban housing prices [29], which causes the agglomerated population to be selected. The “threshold effect” of high housing prices restricts those with low income (less-skilled workers) from congregating in cities with high-speed rails and leads to the accumulation of high-level human capital. 3) Population agglomeration has an effect on the market. High-speed rails improve accessibility to strategic cities and promote population clustering and the expansion of the local market.

Third, high-speed rails drive the agglomeration of innovation capital and innovation-related venture capital within a city. High-speed rails reduce corporate costs, improve market access, and accelerate the agglomeration of innovative enterprises and venture capital, which has high infrastructure requirements [30]. Long et al. (2017) found that high-speed rails facilitate face-to-face communication among venture capital companies and innovative enterprises, reduce the risk from information asymmetries in venture capital decision-making, facilitate the attraction of more venture capital investments to cities and promote innovation [31].

Fourth, high-speed rails create urban specialization; as a result, industrial agglomeration is updated, which further deepens urban innovation [32, 33]. The effect of high-speed railway construction on specialization occurs mainly through industrial transfers. High-speed rails expand the intercity transport network and reduce the time cost of the necessary production factor flows. They also provide incentives for the transfer of industries from cities with higher levels of industrial development to cities with lagging industrial development and accelerate the agglomeration of homogeneous industries within a city [34], thereby promoting specialization. The agglomeration of homogeneous industries can increase the level of urban specialization and effectively promote innovation [35].

High-speed rails not only accelerate the flow of factors but also improve the formation of innovation networks across different cities, which strengthen cooperation in innovation activities among cities on the high-speed rail network, thus improving urban innovation levels [36]. The opening of a high-speed rail optimizes the existing railway transportation network [37], increases the availability of innovative resources from outside the region, and attracts innovative resources due to lower time and economic costs. Better mobility leads to more efficient flows of knowledge and technology within an urban innovation network [38]. In addition, high-speed rails promote the formation of innovation networks characterized by a rapid flow of knowledge and talent across high-speed rail cities. They also accelerate the dissemination of knowledge [39] and improve the efficiency of innovators. Du and Peng (2017) noted that high-speed rails facilitate exchanges and cooperation between entrepreneurs and technologists in different cities connected by high-speed rail [16]. These different cities internalize the knowledge and technology spillovers caused by the flow of high-quality talent, thus improving the innovation level of firms within high-speed rail cities.

High-speed rails also accelerate the dissemination of production knowledge. That is, high-speed rails help enterprises expand the scale and scope of their market, which reduces R&D costs, improves the rate of return on innovation activities, increases innovation incentives, and increases the overall level of innovation within cities. Cai and Ru (2016) found that the market expansion and increasing returns to scale caused by the construction of infrastructure such as high-speed rails have increased the return on innovation investment, stimulated enterprises to initiate more innovation activities, and thus improved the innovation level of high-speed rail cities [40].

Based on the above analysis, we propose the following three hypotheses.

**Hypothesis 1:** High-speed rails promote innovation within cities.

**Hypothesis 2**: High-speed rails promote the agglomeration of innovation factors, including population and investment, within cities, further upgrading those cities’ level of innovation.

**Hypothesis 3**: The opening of a high-speed rail promotes knowledge and technology spillovers within high-speed rail cities and increases the innovation level of cities located on high-speed railways.

## Research design

### Model specification

We treat the opening of high-speed rails in China as a quasi-natural experiment [41] and use a difference-in-differences (DID) model to examine how the opening of a high-speed rail affects urban innovation levels. China’s high-speed railway network has grown since 2008, and new cities have opened high-speed railway nearly every year. Therefore, the traditional DID model is not suitable for evaluating the effect of high-speed rails on innovation. In line with previous studies [42, 43], we construct a multiphase DID model for analysis, as shown in Eq (1):

$$\ln(innovation_{it})=\alpha_{0}+\alpha_{1}{hsr}_{it}+\sum \delta_{k}year_{k}+\sum \gamma_{j}X_{jit}+\mu_{city}+\varepsilon_{it}$$ (1)

In Eq (1), ln(*innovation*_*it*_) indicates the logarithm of the urban innovation level and the coefficient *α*_1_ reflects the effect of the high-speed rail on the urban innovation level. *Year* represents a vector of time dummies, and the corresponding coefficients reflect the time trend in the characteristics of the urban innovation level. *X* is a set of variables that affect the level of urban innovation. *μ*_*city*_ represents the city fixed effects, and *ε*_*it*_ is the error term. By including city fixed effects and year fixed effects, the differences in individual characteristics among cities and city characteristics that vary over time are effectively controlled for.

To verify hypothesis 2 and hypothesis 3, we construct a mediation effect model, as shown in Eqs (2) to (4), to test whether the opening of a high-speed rail can improve city innovation by driving the agglomeration of the population and investment in the city.


$$\ln(innovation_{it})=\beta_{0}+\beta_{1}{hsr}_{it}+\sum \delta_{k}year_{k}+\sum \gamma_{j}X_{jit}+\mu_{city}+\varepsilon_{it}$$ (2)

$$den_{it}=\varphi_{0}+\varphi_{1}{hsr}_{it}+\sum \delta_{k}year_{k}+\sum \gamma_{j}X_{jit}+\mu_{city}+\varepsilon_{it}$$ (3)

$$\ln(innovation_{it})=\theta_{0}+\theta_{1}{hsr}_{it}+\theta_{2}den_{it}+\sum \delta_{k}year_{k}+\sum \gamma_{j}X_{jit}+\mu_{city}+\varepsilon_{it}$$ (4)

In Eq (3), *den*_*it*_ denotes the factor density (population density and investment density). *φ*_1_ indicates the effect of the high-speed rail on the factor density. If *β*_1_ in Eq (2) is statistically significant, then the high-speed rail has a significant impact on urban innovation. Given Eqs (2), (3) and (4), significant values for *φ*_1_ and *θ*_2_ demonstrate that high-speed rail has an indirect impact on urban innovation via the agglomeration of urban factors, and the magnitude of the indirect effect is *φ*_1_×*θ*_2_. If both coefficients *φ*_1_ and *θ*_2_ are not significant at the same time, it means that high-speed railways do not have an indirect impact on urban innovation through an influence on the agglomeration of innovation factors.

To test hypothesis 3, that is, to explore whether high-speed rails promote urban innovation by strengthening the exchange of knowledge and increasing knowledge spillovers within cities connected to the high-speed rail, we further build a spatial lag model and a spatial error model, as shown in Eqs (5) and (6):

$$\ln(innovation_{it})=\alpha_{0}+\rho \sum_{k=1}^{n}w_{ik}\;\ln(innovation_{jt})+\sum \gamma_{j}X_{jit}+\mu_{city}+\varepsilon_{it}$$ (5)

$$\ln(innovation_{it})=\alpha_{0}+\sum \gamma_{j}X_{jit}+\varphi_{it},\;\varphi_{it}=\lambda \sum_{k=1}^{n}w_{ij}\varphi_{it}$$ (6)

where *w* is a spatial weight matrix. We divide the full sample into two groups: observations from before the opening of the high-speed railway and those from after the opening. To meet the estimation requirements for the spatial panel model, the sample is processed as follows: using 2011 as the cutoff year, we delete the subsample of cities that opened a high-speed railway in 2011 or any following year, and we delete the subsamples of all cities that opened a high-speed railway in 2010 or before. In the retained sample from 2011 to 2016, all cities with a high-speed rail had the high-speed rail during the sample period, while the non-high-speed rail cities had not opened a high-speed railway during the sample period. To obtain observations of cities before they opened a high-speed railway, the national railway development plan published in 2004 was considered. This plan inevitably affected the flow of population and investment factors. Therefore, this paper deletes observations from after 2004. No high-speed rail was open in any city in the sample from 2003 to 2004.

The spatial weight matrix represents the interactions among different cities in their innovation activities, namely, the spatial spillovers. To measure the spatial innovation spillovers in high-speed rail cities, we establish a binary weight matrix: we assume that an innovation spillover exists between high-speed rail cities, so we use 1 to indicate the innovation relationships among the high-speed rail cities. We also assume that no innovation spillover exists between non-high-speed rail cities, so we assign a value of 0 to those relationships.

If the estimation coefficients on the spatial terms are significantly positive, then the innovation activities in high-speed rail cities have spillover effects, and hypothesis 3 is correct. If the estimation coefficients on the spatial terms are significantly negative, then high-speed rail cities siphon the innovation activities of other cities, and hypothesis 3 is not correct. If the coefficients on the spatial terms are not statistically significant, then there is no spillover effect in innovation activities between high-speed rail cities, and high-speed rail cities do not siphon the innovation activities of other cities. In this case, hypothesis 3 is still incorrect.

### Variable selection and summary statistics

The measurement of the city innovation level directly determines the reliability of the empirical results in this paper. Currently, the *China City Statistics Yearbook* and the *China Regional Economic Statistics Yearbook* do not disclose innovation indicators at the city level, and most databases collect innovation indicators for only a few large and medium-sized cities in China. The current literature on urban innovation is limited, and Wang et al. (2016) examined only a few large cities in China to determine the impact of creative class clustering on urban innovation [44]. Gao (2015) manually retrieved urban patent data from the China Patent Information Network and tested the impact of city size and human capital on city innovation levels [45]. Manual retrieval of urban patents is time consuming, and the quality and value of the patents are heterogeneous. Conversely, in terms of the agglomeration of innovation factors, allocation of innovation resources, and implementation of innovation policy, significant differences exist between large cities and small or medium-sized cities, and the generality and universality of previous findings need to be further verified. The *China City and Industrial Innovation Report 2017* provides a detailed innovation index for Chinese cities from 2001 to 2016. In the report, a patent renewal model was estimated, and the forecasted market value of patents was aggregated to the city level to construct the urban innovation index. This index relieves the concern regarding the heterogeneity in patent quality and value. Due to this advantage, we used the urban innovation index from this report to measure urban innovation [46].

The opening of a high-speed rail is the core explanatory variable. If city *i* opens a high-speed rail before September of year *t*, then *hsr*_*it*_ = 1; otherwise, *hsr*_*it*_ = 0.

We included the following control variables [47]:

1. *Openness*: The level of foreign direct investment expressed in terms of the ratio of foreign direct investment actually utilized to the GDP of the region.
2. *Top universities dummy*: We consider whether there is a top university in city *i* as an indicator of the quality of the science and education resources in that city. In 1995, the central government of China launched the “211 Project”, which focuses on improving the quality of scientific research in colleges and universities. After years of development, selection into the 211 Project has become an important standard for measuring the quality of science and education in colleges and universities. Based on this, we define the top universities dummy according to whether the city has 211 Project universities.
3. *Human capital*: The share of students in colleges and universities in the total urban population is used as an index to measure the scale of the science and education resources in the city.
4. *GDP per capita*: The level of economic development is expressed as GDP per capita.
5. *Financial development*: For financial development, we use the ratio of bank deposits and loan balances to GDP.
6. *Industrial structure*: The industrial structure is measured as the share of the regional GDP of the nonagricultural sector in the total regional GDP.
7. *Innovation policy*: The strength of government support for innovation is expressed as the share of science and technology expenditure in government financial expenditure.
8. *Entrepreneurial activity*: Entrepreneurial activity is expressed as the share of private and individual urban employees in the total urban population at the end of the year.

Details on the variables and data sources are given in Table 1.

**Table 1:** Variables and data sources.

Variables	Definition	Data Source
*Urban innovation*	Log(innovation index)	*China City and Industrial Innovation Report 2017*
*hsr*	Virtual variable, set according to the opening time of high-speed rail	*Website of China Railway Corporation*
*Treated city with hsr*	Virtual variable, set according to the opening time of high-speed rail	*Website of China Railway Corporation*
*Population density*	Total population/administrative area	*China City Statistical Yearbook*
*Investment density*	Investment scale/administrative area	*China City Statistical Yearbook*
*Opening level*	Foreign direct investment/GDP	*China City Statistical Yearbook*
*Human capital*	Students in colleges and universities/urban population	*China City Statistical Yearbook*
*Top universities dummy*	Virtual variable, set according to the resident setting of top universities	*Website of the Ministry of Education of China*
*GDP per capita*	GDP/urban population	*China City Statistical Yearbook*
*Financial development*	Bank deposit and loan balance/GDP	*China City Statistical Yearbook*
*Industrial structure*	Nonagricultural GDP/GDP	*China City Statistical Yearbook*
*Innovation policy*	S&T expenditure/government financial expenditure	*China City Statistical Yearbook*
*Entrepreneurial activity*	Private and individual employees/urban population	*China City Statistical Yearbook*

Table 2 shows the summary statistics of the variables. The last column shows the correlation coefficients between each variable and the urban innovation level. The correlation coefficients are significantly positive at a confidence level of 99%. Specifically, the opening of a high-speed rail is positively correlated with urban innovation.

**Table 2:** Summary statistics.

Variables	sample size	mean	standard deviation	minimum	maximum	correlation coefficient
*Log(urban innovation)*	3,990	-0.321	1.904	-5.272	6.967	1
*hsr*	3,990	0.159	0.366	0.000	1.000	0.5048***
*Treated city with hsr*	3,990	0.439	0.496	0.000	1.000	0.3711***
*Population density*	3,702	0.422	0.322	0.005	2.648	0.4556***
*Investment density*	3,987	1.036	1.595	0.000	20.421	0.6945***
*Opening level*	3,988	0.029	0.032	0.000	0.454	0.2848***
*Human capital*	3,990	0.015	0.022	0.000	0.131	0.5613***
*Top universities dummy*	3,990	0.133	0.340	0.000	1.000	0.5162***
*GDP per capita*	3,987	8.306	0.785	0.000	11.005	0.7330***
*Financial development*	3,988	2.041	0.975	0.508	8.877	0.4827***
*Industrial structure*	3,990	0.854	0.093	0.000	1.000	0.5791***
*Innovation policy*	3,990	0.011	0.013	0.000	0.207	0.6080***
*Entrepreneurial activity*	3,990	0.101	0.120	0.000	1.524	0.5152***

Note

*, **and *** indicate that the correlation coefficients are significant at confidence levels of 10%, 5% and 1%.

**Fig 1:**
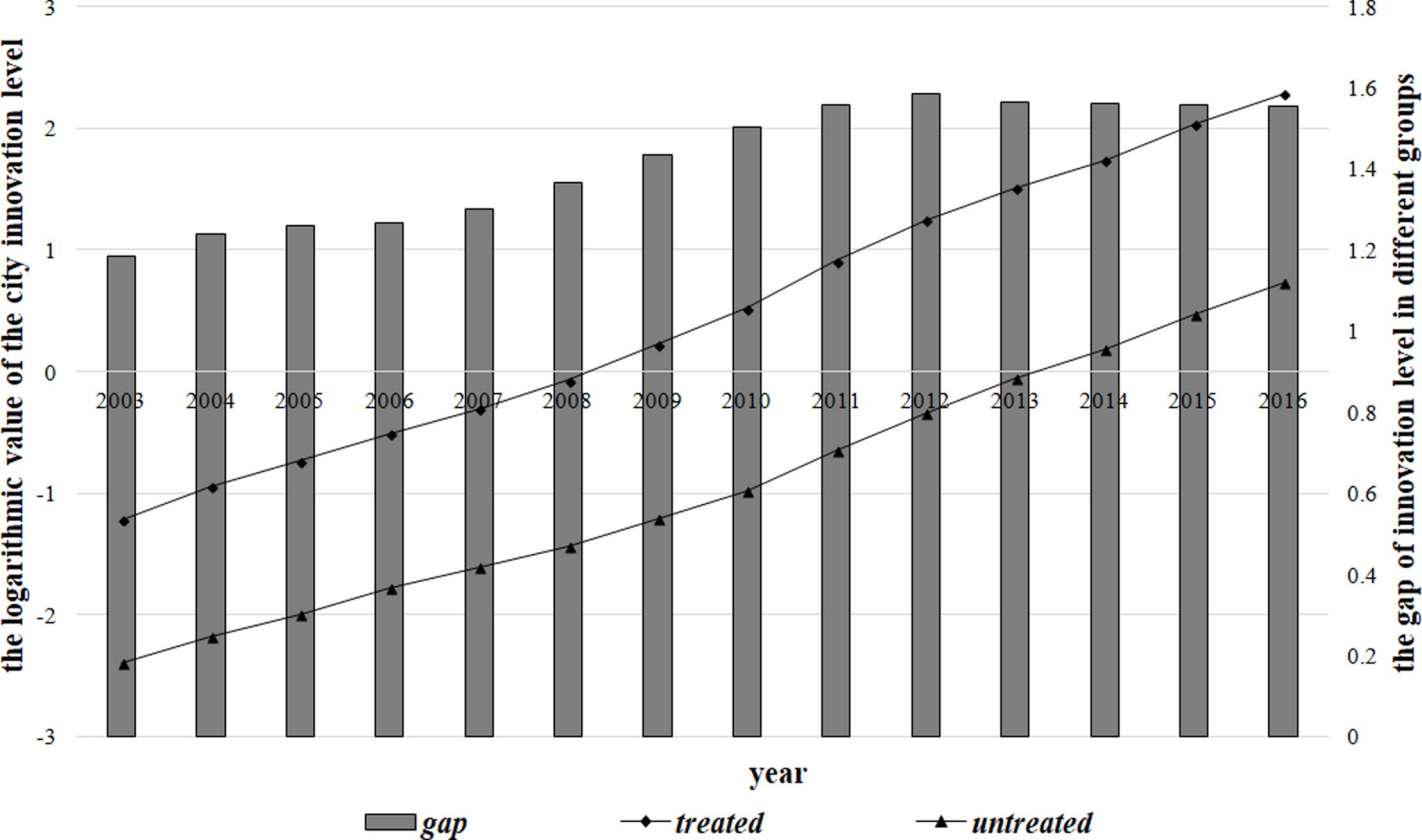
Mean value of the urban innovation level and the trend of innovation difference in China.

In addition, to further explore the impact of high-speed rails on urban innovation, we show the average innovation level of high-speed rail cities and non-high-speed rail cities in each year in Fig 1.

In Fig 1, the abscissa represents the year, and the vertical coordinates represent the logarithmic value of the city innovation level (left) and the difference between the logarithmic value for high-speed rail cities and non-high-speed rail cities (right). The innovation levels of high-speed rail cities and of non-high-speed rail cities seem to have a common trend before 2008; that is, the data satisfy the common trends assumption. Compared with non-high-speed rail cities, high-speed rail cities have a higher level of innovation. Moreover, the difference in the average innovation level between the two groups has increased since 2008, which is the year when China formally began its rapid construction of high-speed rails. Therefore, Fig 1 shows that high-speed rails in China have likely promoted urban innovation. In the following section, we use the DID method to verify this effect.

### Common trends test

The application of DID to policy evaluation requires that the treated group and the control group share a common trend before policy implementation. We use Eq (7) to test whether the innovation levels of the treated group and the control group satisfy the common trend assumption before 2008:

$$\ln(innovation_{it})=\alpha_{0}+\alpha_{1}treated_{i}+\sum_{k=2003}^{2007}\delta_{k}year_{k}+\sum_{k=2003}^{2007}\gamma_{k}year_{k}\cdot treated_{i}+\varepsilon_{it}$$ (7)

*Treated*_*i*_ in Eq (7) is a group dummy variable: for high-speed rail cities, *treated* = 1, and for non-high-speed rail cities, *treated* = 0. *Year* represents the year dummy variables; the time span in Eq (7) is 2003 to 2007. *Year*_*k*_⋅*treated*_*i*_ denotes the interaction term between the *year* dummy and the *group* dummy. If the *γ*_*k*_ coefficients are not significant, then no significant difference exists between the treated group and the control group, and the DID framework is feasible in our context.

In our estimation results, the result of the joint significance test for the *γ*_*k*_ coefficients is *P*(*γ*_1_ = *γ*_2_ = *γ*_3_ = *γ*_4_ = 0) = 0.991. This result indicates that no significant difference exists in the trend in innovation levels between high-speed rail cities and non-high-speed rail cities before the opening of the high-speed railways, which validates the use of the DID framework.

## Empirical analysis

### Benchmark regression

To test the impact of high-speed rails on urban innovation, we estimate Eq (1). The results are shown in Table 3. Model 1 includes the dummy variable for high-speed rail operation only. The coefficient is significantly positive, which shows that after the opening of a high-speed rail, urban innovation increases significantly. There are three possible reasons. The first is a time trend effect: urban innovation is increasing from year to year. The second is selection bias: cities with high-speed rails have intrinsically high innovation levels. The third possible reason is that urban innovation increases significantly due to the opening of the high-speed rail.

**Table 3:** Benchmark regression.

	Model 1	Model 2	Model 3	Model 4	Model 5	Model 6
*hsr*	2.627***	1.738***	0.778***	0.208***	0.251***	0.110***
	(0.071)	(0.073)	(0.083)	(0.024)	(0.056)	(0.022)
*Treated city with hsr*			1.142***		0.594***	
			(0.054)		(0.038)	
*Opening level*					0.795	-0.813***
					(0.536)	(0.310)
*Human capital*					4.285***	-2.386**
					(1.077)	(0.996)
*Top universities dummy*					1.398***	
					(0.069)	
*GDP per capita*					0.406***	0.138***
					(0.044)	(0.026)
*Financial development*					0.113***	0.097***
					(0.023)	(0.018)
*Industrial structure*					2.199***	2.056***
					(0.264)	(0.243)
*Innovation policy*					29.828***	15.352***
					(1.595)	(0.743)
*Entrepreneurial activity*					1.026***	0.920***
					(0.162)	(0.121)
*Constant*	-0.740***	-1.880***	-2.381***	-1.880***	-8.257***	-2.593***
	(0.028)	(0.088)	(0.087)	(0.022)	(0.320)	(0.276)
Time fixed effects	NO	YES	YES	YES	YES	YES
City fixed effects	NO	NO	NO	YES	NO	YES
Sample size	3990	3990	3990	3990	3985	3985
*R* ^2^	0.255	0.387	0.449	0.892	0.757	0.910

Note

*, ** and *** indicate that the regression coefficients are significant at confidence levels of 10%, 5% and 1%, respectively.

Building on model 1, model 2 includes time dummy variables, which control for the time trend described in the first reason above. The results show that the coefficient on the high-speed rail variable remains significantly positive, and the results for the time dummy variables show that city innovation levels increase year over year (due to space limitations, we omit the results for the time dummy variables from Table 2).

Model 3 further includes the group dummy variable. The results show that the coefficient on the group dummy variable is significantly positive, which indicates that there is some selection bias in the opening of high-speed railways. After controlling for the differences between the treated group and the control group, the coefficient on the high-speed rail variable is still significantly positive, which indicates that the opening of high-speed rails significantly improves urban innovation levels.

Model 4 further includes city fixed effects. Model 5 and Model 6 add more control variables that affect urban innovation. The results continue to show that the coefficient on the high-speed rail variable is significantly positive, which indicates that the opening of a high-speed rail can significantly increase urban innovation. As a result, hypothesis 1 is confirmed.

### Robustness

#### Robustness test I: PSM-DID

As previously noted, compared with non-high-speed rail cities, high-speed rail cities have an inherently high level of innovation. This potential selection bias could contaminate our estimation results. To ease this concern, we first created matched samples using propensity score matching, and then we re-estimated the DID model using the matched samples. We used the control variables as the matching variables and used one-to-one nearest neighbor matching (without replacement). We obtained a sample of 3500 cities after matching, with 1750 cities in the treated group and 1750 cities in the control group. The results are shown as Model 1 in Table 4. We can see that the coefficient on the high-speed rail variable is significantly positive, which demonstrates that high-speed rails significantly improve the urban innovation level, so we need not be concerned about selection bias.

**Table 4:** Robustness test.

	Model 1	Model 2	Model 3	Model 4
	PSM-DID	DID single time point	Alternative measure	Placebo test
*hsr*	0.044**	0.216***	3.715***	0.007
	(0.022)	(0.029)	(0.268)	(0.022)
*Constant*	1.139***	-2.876***	21.711***	2.138***
	(0.430)	(0.283)	(3.176)	(0.738)
Control variables	YES	YES	YES	YES
Time fixed effects	YES	YES	YES	YES
City fixed effects	YES	YES	YES	YES
Sample size	3500	3075	3622	1420
*R* ^*2*^	0.921	0.91	0.391	0.987

Note

*, ** and *** indicate that the regression coefficients are significant at confidence levels of 10%, 5% and 1%, respectively.

#### Robustness test II: DID using an alternative cutoff year

In line with previous studies [47], we altered the cutoff year for the opening of a high-speed rail to 2011. Cities that opened high-speed railways in 2011 or before became our treated group, and other cities became our control group. To reduce the impact of the sample of cities with a high-speed rail built after 2011 on the results, we deleted those cities that opened their high-speed railway after 2011 and used a traditional DID model to test the robustness of our results. The results are shown in model 2 in Table 4. The coefficient on *hst*_*it*_ is significantly positive, which indicates that the opening of a high-speed rail promotes urban innovation.

#### Robustness test III: Alternative indicators for city innovation level

Patents are the most direct output of innovation activities; previous studies have used patent output as an indicator for the level of innovation in an area [48]. As a robustness check, we use the annual number of patent applications at the city level as an index to measure urban innovation. The data are from the patent cloud database (https://www.patentcloud.net). The results are shown in model 3 in Table 4. The coefficient on the high-speed rail variable is significantly positive, which shows that the opening of high-speed rails promotes urban patent applications and once again confirms the robustness of our results.

#### Robustness test IV: Placebo test

When using the DID model to test the impact of high-speed rails on urban innovation, the assumption is that if opening a high-speed rail has no impact, there will be no systematic difference in the trends in the innovation level between high-speed rail cities and non-high-speed rail cities [8]. We use a counterfactual analysis to test our previous findings. Specifically, we first delete the data for cities in the year when the first high-speed railway opened in China and all subsequent years (i.e., 2008 and later). Then we assume that the first high-speed railway opened in 2005 and create the corresponding dummy to indicate high-speed rail cities and the corresponding interaction term. We re-estimate the DID model; the results are shown in Model 4 of Table 4. The coefficient on *hsr* is not statistically significant, indicating that there is no systematic difference in the trends in innovation levels between high-speed rail cities and non-high-speed rail cities due to the opening of a high-speed rail. This confirms our previous findings.

### Spatial heterogeneity

In general, significant differences exist among cities in terms of their ability to attract innovation resources, their ability to adopt innovation policy, and the efficiency with which they utilize innovation factors, which may cause spatial heterogeneity in the effect of high-speed railways on innovation. Therefore, we further divide the sample of high-speed rail cities into cities with a higher level of science and education, cities with a lower level of science and education, core cities (such as provincial capitals, municipalities directly under the supervision of the central government, and subprovincial cities), and prefecture-level cities as well as cities in the eastern, central, or western region to explore the spatial heterogeneity of the innovation effect of high-speed rail from the heterogeneity of science and education level, administrative level heterogeneity and location heterogeneity.

First, to analyze the effect of opening high-speed railways on innovation in cities with different levels of science and education, we group the high-speed rail cities into cities with a higher level of science and education and cities with a lower level. Colleges and universities are important bases for the training of personnel and for scientific research activities and can provide good scientific and educational resources for innovation. They are a key subject in China’s innovation activities. Cities with 211 Project universities are defined as having a higher level of science and education; cities without 211 Project universities are defined as having a lower level of science and education. The results are shown in model 1 and model 2 in Table 5. In model 1, the impact of high-speed rails on city innovation is positive but not statistically significant. In model 2, the coefficient on high-speed rails is significantly positive. The results demonstrate that the opening of a high-speed railway significantly improves innovation levels in cities with a lower level of science and education but has no significant impact on the innovation level of cities with a higher level.

**Table 5:** Spatial heterogeneity analysis.

	Model 1	Model 2	Model 3	Model 4	Model 5	Model 6
	Heterogeneity in science and education development		Heterogeneity in city development		Heterogeneity in geographical location	
	High level	Low level	Core cities	prefecture-level cities	Eastern cities	Central & western cities
*hsr*	0.118	0.280***	0.094	0.289***	0.368***	0.144*
	(0.113)	(0.062)	(0.113)	(0.062)	(0.079)	(0.079)
*Constant*	-8.567***	-8.323***	-8.406***	-8.503***	-9.091***	-8.591***
	(0.378)	(0.332)	(0.375)	(0.333)	(0.348)	(0.389)
Control variables	YES	YES	YES	YES	YES	YES
Time fixed effects	YES	YES	YES	YES	YES	YES
City fixed effects	YES	YES	YES	YES	YES	YES
Sample size	2612	3594	2599	3607	3061	3145
*R* ^*2*^	0.763	0.659	0.767	0.652	0.743	0.636

Note

*, ** and *** indicate that the regression coefficients are significant at confidence levels of 10%, 5% and 1%, respectively.

Second, to test the impact of high-speed rails on the innovation levels of cities with different administrative levels, we further categorize the high-speed rail cities into core cities and prefecture-level cities. We compare these two groups of high-speed rail cities with non-high-speed rail cities; the results are shown in model 3 and model 4 in Table 5. The coefficient for the effect of the high-speed railway variable on the innovation level of the core cities is not significant. In normal prefecture-level cities, the coefficient is significantly positive. These results show that the opening of a high-speed rail can increase urban innovation in prefecture-level cities but has no significant impact on the innovation level of core cities.

Observing the results for the above two groups, we find that the opening of a high-speed railway can improve the innovation level of cities with a lower level of science and education and of general prefecture-level cities but has no significant impact on cities with a higher level of science and education or on core cities. This may be because prefecture-level cities and cities with a lower level of science and education have a latecomer advantage that core cities and cities with a higher level of science and education do not have. The opening of a high-speed rail and the effect of the accumulation of innovation factors and the knowledge spillovers brought about by the high-speed rail can effectively tap into the innovation potential of these cities and significantly increase their level of innovation. In core cities and cities with a higher level of science and education, innovation activities are already taking place at a higher level, or these cities may even be on the leading frontier of innovation in China; hence, the opening of a high-speed rail has little or no significant marginal effect on urban innovation.

Finally, we categorize the high-speed rail cities into “eastern cities” (including Beijing, Tianjin, Shanghai, and cities in provinces of Hebei, Liaoning, Shandong, Jiangsu, Zhejiang, Fujian, Guangdong and Hainan) and “central and western cities” (including Chongqing and cities in the provinces of Shanxi, Inner Mongolia, Jilin, Heilongjiang, Anhui, Jiangxi, Henan, Hubei, Hunan, Guangxi, Sichuan, Shaanxi, Guizhou, Yunnan, Tibet, Gansu, Qinghai, Ningxia and Xinjiang) according to their geographical location; the results are shown in model 5 and model 6 in Table 5. For the eastern region, the coefficient for the effect of the opening of a high-speed railway on urban innovation is significantly positive, while for the central and western regions, the coefficient is significant at the 90% confidence level and is positive. The opening of a high-speed rail significantly improves urban innovation levels regardless of geographical location. To summarize, differences in location do not affect the relation between innovation and the opening of high-speed railways.

## Transmission mechanism

### Population agglomeration

People play the most active role in innovation activities. The opening of high-speed railways may stimulate the agglomeration of the urban population, hence promoting urban innovation. To verify this proposed mechanism, we estimate Eq (2), Eq (3) and Eq (4); the results are shown in model 1, model 2 and model 3, respectively, in Table 6. The coefficient on the high-speed railway variable in model 1 is significantly positive, which demonstrates that the opening of a high-speed rail promotes urban innovation. Model 2 shows that the coefficient for the effect of high-speed rails on urban population density is significantly positive. This result indicates that the opening of high-speed rails increases the population density within cities and accelerates the agglomeration of the population. In model 3, the coefficient for the impact of population density on the level of urban innovation is also significantly positive, which shows that population agglomeration can effectively improve the level of urban innovation.

**Table 6:** Transmission pathway: Mediation effect model.

Mediation Variable	Population Agglomeration			Investment Agglomeration		
Model	Model 1	Model 2	Model 3	Model 4	Model 5	Model 6
Dependent variable	*Log(urban innovation)*	*Urban population density*	*Log(urban innovation)*	*Log(urban innovation)*	*Urban investment density*	*Log(urban innovation)*
*hsr*	0.113***	0.021***	0.101***	0.110***	0.616***	0.051**
	(0.023)	(0.002)	(0.023)	(0.022)	(0.039)	(0.023)
*Density (pop or inv)*			0.557***			0.095***
			(0.202)			(0.009)
*Constant*	1.929***	0.734***	1.520***	1.801***	5.602***	1.266***
	(0.363)	(0.031)	(0.392)	(0.360)	(0.625)	(0.359)
Control variables	YES	YES	YES	YES	YES	YES
Time fixed effects	YES	YES	YES	YES	YES	YES
City fixed effects	YES	YES	YES	YES	YES	YES
Sobel test	0.012(z = 2.675, p = 0.007)			0.059(z = 8.568, p = 0.000)		
Sample size	3697			3982		
*R* ^*2*^	0.971	0.993	0.971	0.970	0.871	0.971

Note

*, ** and *** indicate that the regression coefficients are significant at confidence levels of 10%, 5% and 1%, respectively.

Combining the results of model 2 and model 3 suggests that the opening of a high-speed rail can accelerate population agglomeration and indirectly promote urban innovation. The results of the Sobel test also suggest that population agglomeration has a mediating effect. After controlling for the mediating effect of population agglomeration, the coefficient for the influence of high-speed rails on urban innovation in model 3 remains significantly positive, which shows that high-speed rails continue to play a significant role in increasing urban innovation even after controlling for the mediating effect of population agglomeration.

### Investment agglomeration

In addition to population agglomeration, the opening of a high-speed rail increases urban investment, which is also an important mechanism by which high-speed rails promote urban innovation. To test the validity of this mechanism, we utilize the density of the city’s investment in fixed assets as a mediating variable and estimate regressions that take the form of Eqs (2) to (4). The results are shown in model 4 to model 6 in Table 6.

Model 4 shows that the opening of a high-speed rail plays a significant role in promoting urban innovation. Further investigation in model 5, which estimates the impact of high-speed rails on the density of city investments in fixed assets, shows that the coefficient is significantly positive, indicating that the opening of a high-speed rail significantly increases the density of city investments in fixed assets. In model 6, the coefficient for the effect of the density of city investments in fixed assets on urban innovation is significantly positive, indicating that such investment significantly promotes urban innovation. Models 5 and 6 imply that the opening of high-speed rails can significantly increase the density of city investments in fixed assets, thereby playing a significant role in promoting urban innovation. The Sobel test results also support the existence of this intermediary effect.

After controlling for the mediating effect of investment agglomeration, the coefficient indicating the effect of high-speed railways on the level of urban innovation in model 6 remains significantly positive. This result indicates that after controlling for the mediating effect of investments in fixed assets by cities, the opening of a high-speed rail continues to play a significant role in promoting urban innovation.

To further test whether the regression results regarding the mediating effects noted above are significant, we use a bootstrap test to verify the above conclusions. The results are shown in Table 7.

**Table 7:** Bootstrap test.

Mediation variable	*Urban Population Density*	*Urban Fixed Investment Density*
Bootstrap test	0.012	0.059
(mediation effect)	(z = 2.85, p = 0.004)	(z = 4.94, p = 0.000)
Mediation effect proportion	10.22%	53.40%
Bootstrap test	0.101	0.051
(direct effect)	(z = 3.41, p = 0.001)	(z = 2.05, p = 0.040)
Direct effect proportion	89.78%	46.60%
Total effect	0.113	0.110
	(z = 4.89, p = 0.000)	(z = 4.93, p = 0.000)

The total effect, the mediating effect and the indirect effect of the opening of high-speed rails on urban innovation are significantly positive at a higher level of confidence. In summary, the opening of high-speed rails significantly promotes urban innovation. On the one hand, human capital plays a core role in innovation. The opening of high-speed rails leads to a greater concentration of human capital; hence, it facilitates innovation. On the other hand, investment in capital and fixed assets is indispensable for innovative activities. The opening of high-speed rails increases the density of city investments in fixed assets and thus plays a significant indirect role in promoting urban innovation.

Of these results, the mediating effect of population agglomeration explains approximately 10.22% of the total effect, while the mediating effect of investment agglomeration explains approximately 53.40% of the total effect. Combined, these two mediators explain approximately 63% of the total effect. Urban population agglomeration and investment agglomeration driven by the opening of high-speed rails are the main reasons for the improvement in the urban innovation level. The above conclusion confirms hypothesis 2.

### Spillover effect of innovation activities

The reason that the opening of a high-speed rail can promote urban innovation is likely that it facilitates travel by entrepreneurs and scientific researchers, enabling them to have face-to-face exchanges, and it increases the exchange of knowledge and knowledge spillovers due to the flow of researchers and innovative products. Therefore, to test whether the opening of high-speed rails increases knowledge exchanges and spillovers across different cities (i.e., to test whether hypothesis 3 is valid), we estimate the spatial panel models described by Eqs (5) and (6). The results are shown in Table 8.

**Table 8:** Transmission mechanism: Spatial panel model.

	(1)	(2)	(3)	(4)
	Before the opening of high-speed railway		After the opening of high-speed railway	
*W· Log(urban innovation)*	0.002		0.005***	
	(0.004)		(0.000)	
*W·e*		0.001		0.004***
		(0.001)		(0.001)
*Constant*	-2.225	-3.022	-4.725***	-2.752***
	(2.729)	(2.370)	(0.590)	(0.430)
Control variables	YES	YES	YES	YES
City fixed effects	YES	YES	YES	YES
LR Test	0.188	0.973	167.779***	26.175***
N	440	440	1320	1320
R^2^	0.960	0.968	0.917	0.923

Note

*, ** and *** indicate that the regression coefficients are significant at confidence levels of 10%, 5% and 1%, respectively.

Columns (1) and (2) in Table 8 present the correlation between innovation and being a high-speed rail city before the high-speed rail is opened. Regression (1) presents the results of the spatial panel lag model, and regression (2) presents the results of the spatial panel error model. We can see that the LR test accepts the null hypothesis that the coefficient of the spatial term is 0 compared with the ordinary least square regression model. In column (1) and column (2), the coefficients on the spatial terms are not statistically significant. The above results show that no spatial correlation or spillover effect of innovation was present in high-speed rail cities before the opening of the corresponding high-speed rails.

Columns (3) and (4) in Table 8 present the results of the spillover effects of the high-speed rail on innovation in high-speed rail cities after the high-speed rail is opened. The LR test implies that the null hypothesis that the coefficient on the spatial term is 0 should be rejected, which indicates that the model has a spatial effect. The coefficient on the spatial lag of the dependent variable is significantly positive, which indicates that an improvement in the innovation level of a high-speed rail city significantly increases innovation within other high-speed rail cities. A significant innovation spillover effect within high-speed rail cities can be observed; hypothesis 3 is confirmed. The intensification of the innovation spillover effect in high-speed rail cities can explain the impact of high-speed rails on the spatial pattern of urban innovation. The opening of a high-speed rail promotes knowledge spillovers among high-speed rail cities and not only widens the gap in innovation levels between high-speed rail cities and non-high-speed rail cities but also narrows the gap in innovation across high-speed rail cities, indicating the presence of a "club effect".

## Discussion

The development of high-speed rails has provided China with an outstanding transportation infrastructure that acts as a foundation for urban innovation and development and promotes urban scientific and technological innovation. Based on data covering 285 cities in China from 2003 to 2016, we empirically examined the impact of the opening of high-speed railways on urban innovation and the spatial heterogeneity in that impact. We found that the opening of a high-speed rail significantly improves the urban innovation level, and the robustness tests (the PSM-DID method, the single time-point DID model, the alternative measure of urban innovation, and the placebo test) showed that the above conclusions are robust. Our main results are similar to those from the studies of Zeng et al. (2021) and Lin (2017), who found that the opening of high-speed rails can promote urban innovation [7, 9]. However, in contrast with their research, we use a city innovation index, which takes into account the heterogeneity in patent value, to measure levels of urban innovation. This index alleviates the measurement error in the urban innovation level caused by differences in the value of patents of different ages, so our conclusions are more robust than those of the abovementioned studies. Of course, we also use patent output to measure the level of innovation and draw conclusions consistent with those of the above studies.

This conclusion is not surprising and is consistent with our hypothesis. Transport infrastructure is an important part of urban innovation systems. The improvement of transportation infrastructure can improve the convenience and efficiency of travel, thus attracting more innovation factors and laying a foundation for innovation activities. In addition, the improvement of transportation infrastructure accelerates the dissemination of knowledge and technology, thus promoting urban innovation. This is also the conjectured mechanism by which the opening of a high-speed rail promotes urban innovation, as proposed in hypothesis 2 and hypothesis 3.

We explore the mechanisms underlying the impact of high-speed rails on urban innovation, and our mechanism differs from that of Zeng et al. (2021) and Lin (2017) [7, 9]. They examined R&D scale, foreign trade, market competition and industrial agglomeration as potential mechanisms. Through the use of a mediating effect model, we examine the effect of the opening of high-speed rails on innovation as a result of innovation factor agglomeration and knowledge spillover. This perspective is also the main contribution of our research. Specifically, on the one hand, the opening of high-speed rails has accelerated the agglomeration of population and investment and has indirectly improved urban innovation. On the other hand, the knowledge spillovers caused by the opening of high-speed railways also provide an important foundation for improvements in the capacity of cities to innovate.

We further test the spatial heterogeneity in the impact of high-speed rails on urban innovation through subsample estimations. The results can facilitate traffic planning by the government according to local conditions and local innovation policy. The positive effect of the opening of a high-speed rail on innovation exhibits significant spatial heterogeneity. High-speed rail openings increase the innovation level in cities with a lower level of science and education and in prefectural-level cities. However, such openings have no significant effect on innovation level improvements in cities with a higher level of science and education, provincial capitals, municipalities directly under the supervision of the central government or subprovincial cities. In addition, the geographic heterogeneity in the impact of high-speed rails on urban innovation is not statistically significant. That is, in both the eastern region and the central and western regions, the opening of a high-speed rail plays a significant role in improving the urban innovation level. Therefore, for cities with low levels of science and education and noncore cities, the government should strengthen the construction of transportation infrastructure and fully utilize infrastructure to promote urban innovation.

## Conclusion and insights

Overall, the opening of high-speed rails can promote urban innovation. This conclusion has passed our robustness tests. The mechanism analysis shows that the opening of high-speed rails can promote urban innovation by accelerating the agglomeration of population and investment and by promoting knowledge spillovers. In addition, high-speed rails promote urban innovation in cities with a lower level of science and education and in prefectural-level cities. However, high-speed rails have no significant effect on urban innovation in cities with a higher level of science and education, provincial capitals, municipalities directly under the supervision of the central government or subprovincial cities. In addition, the geographic heterogeneity in the impact of high-speed rails on urban innovation is not statistically significant.

Our discoveries not only supplement the exploration of the economic effects of the opening of high-speed railways but are also of great policy significance. Moreover, these findings offer important practical insights for countries that aim to implement innovation-driven development and to improve the quality of economic growth. The positive effect of high-speed railway openings on urban innovation is an important way in which railway construction can be used to promote economic growth.

Our lesson is that the government should be aware of the importance of high-speed rails and their stimulating effect on China’s scientific and technological innovation system. Moreover, high-speed rails have profoundly affected regional innovation networks and the pattern of innovation in China. The government should remain vigilant against any "innovation club" effect caused by the high-speed rail network. The opening of high-speed rails has significantly increased the innovation level of high-speed rail cities but has had a minimal impact on non-high-speed rail cities; it may even have accelerated the siphoning of talent and resources out of non-high-speed rail cities into high-speed rail cities. Regarding policy recommendations, we suggest that policymakers should be alert to the divergent trends in the development of innovation between high-speed rail cities and non-high-speed rail cities. It would be wise for policymakers to widen the coverage of the high-speed rail network to cultivate innovation networks that utilize high-speed rails as their carrier and to promote the dissemination and sharing of knowledge and technology.

There are some shortcomings to our article. On the one hand, limited by the urban innovation index data, our sample for empirical analysis can extend only to 2016. The *China City and Industrial Innovation Report* has not been updated, and the data it disclosed cover only up to 2016, so our data sample ranges from 2003 to 2016. It is highly important to observe the effects of the opening of high-speed rails on innovation in more recent years. On the other hand, there is a lack of research on the microfoundations of the effects of high-speed rails on innovation. Macroeconomic phenomena have a microfoundation, so the impact of high-speed rails on company-level innovation is a problem that needs to be studied in the future.
